# Increased Severe Cases and New-Onset Type 1 Diabetes Among Children Presenting With Diabetic Ketoacidosis During First Year of COVID-19 Pandemic in Turkey

**DOI:** 10.3389/fped.2022.926013

**Published:** 2022-06-29

**Authors:** Eylem Kiral, Birgul Kirel, Merve Havan, Mehmet Keskin, Murat Karaoglan, Ahmet Yildirim, Murat Kangin, Mehmet Nur Talay, Tuba Urun, Umit Altug, Selman Kesici, Erennur Tufan, Ebru Kacmaz, Gurkan Bozan, Ebru Azapagasi, Mutlu Uysal Yazici, Zeynelabidin Ozturk, Osman Yesilbas, Gulay Karaguzel, Gulay Kaya, Ulkem Barlas, Muhterem Duyu, Merve Boyraz, Esra Sevketoglu, Nihal Akcay, Suna Hancili, Ayla Guven, Oǧuz Dursun, Nazan Ulgen Tekerek, Gokçen Ozcifci, Pinar Yazici, Eda Turanli, Tanil Kendirli, Fevzi Kahveci, Ayse Filiz Yetimakman, Agop Citak, Guntulu Şik, Ibrahim Bingol, Fatih Aygun, Cansu Durak, Resul Yilmaz, Fuat Bugrul, Yusuf Sari, Hakan Tekguç, Hatice Albayrak, Nazik Yener, Hasan Agin, Ekin Soydan, Dincer Yildizdas, Semine Ozdemir Dilek, Nilufer Yalindag, Feyza Incekoy-Girgin, Nuri Alacakir, Filiz Tutunculer, Mehmet Ozgur Arslanaoglu, Can Aydin, Muzaffer Bilgin, Enver Simsek, Ener Cagri Dinleyici

**Affiliations:** ^1^Pediatric Intensive Care Unit, Eskisehir Osmangazi University Faculty of Medicine, Eskisehir, Turkey; ^2^Department of Pediatric Endocrinology, Eskisehir Osmangazi University Faculty of Medicine, Eskisehir, Turkey; ^3^Pediatric Intensive Care Unit, Mersin City Hospital, University of Health Sciences, Mersin, Turkey; ^4^Department of Pediatric Endocrinology, Gaziantep University Faculty of Medicine, Gaziantep, Turkey; ^5^Pediatric Intensive Care Unit, Gazi Yasargil Training and Research Hospital, University of Heath Sciences, Diyarbakir, Turkey; ^6^Pediatric Intensive Care Unit, Sanliurfa Training and Research Hospital, University of Heath Sciences, Sanliurfa, Turkey; ^7^Pediatric Intensive Care Unit, Hacettepe University Faculty of Medicine, Ankara, Turkey; ^8^Pediatric Intensive Care Unit, Dr Sami Ulus Gynecology Obstetrics and Child Health and Diseases Training and Research Hospital, Ankara, Turkey; ^9^Pediatric Intensive Care Unit, Faculty of Medicine, Karadeniz Technical University, Trabzon, Turkey; ^10^Department of Pediatric Endocrinology, Faculty of Medicine, Karadeniz Technical Universit, Trabzon, Turkey; ^11^Pediatric Intensive Care Unit, Bagcilar Training and Research Hospital, University of Health Sciences, Istanbul, Turkey; ^12^Pediatric Intensive Care Unit, Prof. Dr. Süleyman Yalcin City Hospital, Medeniyet University, Istanbul, Turkey; ^13^Pediatric Intensive Care Unit, Bakirköy Dr Sadi Konuk Research and Training Hospital, University of Heath Sciences, Istanbul, Turkey; ^14^Department of Pediatric Endocrinology, Zeynep Kamil Women and Children Diseases Traning and Research Hospital, University of Heath Sciences, Istanbul, Turkey; ^15^Pediatric Intensive Care Unit, Akdeniz University Faculty of Medicine, Antalya, Turkey; ^16^Van Training and Research Hospital, University of Heath Sciences, Van, Turkey; ^17^Pediatric Intensive Care Unit, Ege University Faculty of Medicine, Izmir, Turkey; ^18^Pediatric Intensive Care Unit, Ankara University Faculty of Medicine, Ankara, Turkey; ^19^Pediatric Intensive Care Unit, Kocaeli University Faculty of Medicine, Kocaeli, Turkey; ^20^Pediatric Intensive Care Unit, Faculty of Medicine, Acibadem Mehmet Ali Aydinlar University, Istanbul, Turkey; ^21^Pediatric Intensive Care Unit, Istanbul University Cerrahpasa Medical Faculty, Istanbul, Turkey; ^22^Pediatric Intensive Care Unit, Selcuk University Faculty of Medicine, Konya, Turkey; ^23^Department of Pediatric Endocrinology, Selcuk University Faculty of Medicine, Konya, Turkey; ^24^Pediatric Intensive Care Unit, Firat University Faculty of Medicine, Elazig, Turkey; ^25^Pediatric Intensive Care Unit, Dr. Burhan Nalbantoglu Hospital, Lefkosa, Cyprus; ^26^Pediatric Intensive Care Unit, Ondokuz Mayis University Faculty of Medicine, Samsun, Turkey; ^27^Pediatric Intensive Care Unit, Dr Behçet Uz Children's Hospital, Izmir, Turkey; ^28^Pediatric Intensive Care Unit, Cukurova University Faculty of Medicine, Adana, Turkey; ^29^Department of Pediatric Endocrinology, Cukurova University Faculty of Medicine, Adana, Turkey; ^30^Pediatric Intensive Care Unit, Marmara University Faculty of Medicine, Istanbul, Turkey; ^31^Pediatric Intensive Care Unit, Trakya University Faculty of Medicine, Edirne, Turkey; ^32^Department of Pediatric Endocrinology, Trakya University Faculty of Medicine, Edirne, Turkey; ^33^Department of Biostatistics, Eskisehir Osmangazi University Faculty of Medicine, Eskisehir, Turkey

**Keywords:** COVID-19, children, diabetes, diabetic ketoacidosis, pandemic, pediatric intensive care unit

## Abstract

**Introduction:**

There have been some significant changes regarding healthcare utilization during the COVID-19 pandemic. Majority of the reports about the impact of the COVID-19 pandemic on diabetes care are from the first wave of the pandemic. We aim to evaluate the potential effects of the COVID-19 pandemic on the severity of diabetic ketoacidosis (DKA) and new onset Type 1 diabetes presenting with DKA, and also evaluate children with DKA and acute COVID-19 infection.

**Methods:**

This is a retrospective multi-center study among 997 children and adolescents with type 1 diabetes who were admitted with DKA to 27 pediatric intensive care units in Turkey between the first year of pandemic and pre-pandemic year.

**Results:**

The percentage of children with new-onset Type 1 diabetes presenting with DKA was higher during the COVID-19 pandemic (*p* < 0.0001). The incidence of severe DKA was also higher during the COVID-19 pandemic (*p* < 0.0001) and also higher among children with new onset Type 1 diabetes (*p* < 0.0001). HbA1c levels, duration of insulin infusion, and length of PICU stay were significantly higher/longer during the pandemic period. Eleven patients tested positive for SARS-CoV-2, eight were positive for new onset Type 1 diabetes, and nine tested positive for severe DKA at admission.

**Discussion:**

The frequency of new onset of Type 1 diabetes and severe cases among children with DKA during the first year of the COVID-19 pandemic. Furthermore, the cause of the increased severe presentation might be related to restrictions related to the pandemic; however, need to evaluate the potential effects of SARS-CoV-2 on the increased percentage of new onset Type 1 diabetes.

## Introduction

Coronavirus disease 2019 (COVID-19), caused by the severe acute respiratory syndrome coronavirus 2 (SARS-CoV-2), is a global pandemic and more than six million individuals died in May 2022, wherein the number of cases in the world surpassed 518 million (1). When compared to adult age groups, the frequency, complications and mortality are lower in children (2–4). While the impact of COVID-19 differs by country, societies are attempting to reduce the utilization of healthcare facilities by limiting the virus's spread and lowering virus-related morbidity and mortality rates (5). In terms of the Stringency Index, the healthcare background of countries and the actual number of cases requiring hospitalization and intensive care units; have an impact on the routine care of chronic disease. During the pandemic, healthcare utilization and pediatric emergency-room visits were said to have declined, prompting worries about the possible underdiagnoses of other medical disorders (6, 7). Furthermore, even in life-threatening situations, patients and parents put off calling healthcare practitioners because they (or their children) are terrified of contracting SARS-CoV-2. The first case of COVID-19 was discovered in Turkey on March 11, 2020. Ministry of Health, Turkey, was declared a public health emergency, prompting the implementation of school closures and governmental regulations aimed at limiting the virus's transmission through social isolation. In particular, from October 2020 to January 2021, a higher number of cases with COVID-19 have been reported during the first year of the pandemic. According to the amount of actual COVID-19 instances in Turkey, there are certain limits and reducing restrictions. The majority of schools and universities were closed during the first year of the pandemic (continued via online education), and long-term serious curfews for children under the age of 18 were adopted as a control strategy (8).

During the pandemic, children who developed COVID-19 non-related disorders, such as diabetes mellitus (DM), had an increased risk of suffering adverse consequences (9). Diabetic ketoacidosis (DKA) is one of the life-threatening complications of diabetes in children that can cause major morbidity (10). In comparison to the early 2010s, the prevalence of DKA with the initiation of Type 1 diabetes has grown in recent years, and younger patients are at a higher risk than school-aged children and adolescents (11). In the United States, 2.7% of children hospitalized with COVID-19 had a history of diabetes, and 2.9% developed DKA during their stay (12). The influence of the COVID-19 pandemic on pediatric diabetes care has been recorded wih an increase in the number of children with severe DKA at admission at first part of the pandemic (7, 13–16). The aim of this study was to evaluate the potential effects of the COVID-19 pandemic on the severity of diabetic ketoacidosis (DKA) and new onset Type 1 diabetes presenting with DKA, and also evaluate children with DKA and acute COVID-19 infection.

## Materials and Methods

This is a multi-centric retrospective study population that included children less than 18 years of age admitted to pediatric intensive care units in Turkey presenting with DKA. Data were collected from 27 pediatric intensive care units from March 1, 2019 to February 28, 2021. The COVID-19 pandemic group comprised those presenting with DKA from March 11, 2020 (the first case reported in Turkey) to February 28, 2021. The pre-pandemic group included those diagnosed with DKA from March 1, 2019 to February 29, 2020.

Ethics approval was obtained from the Eskisehir Osmangazi University Local Ethical Committee (26 January 2021, numbered E-25403353-050.99-146275). This study has also been approved by the Ministry of Health. This study is a retrospective medical records review; our study did not require written informed consent, and none of the personally identifiable information has been shown in the study.

The coordinating center (Eskisehir Osmangazi University Faculty of Medicine) sent a questionnaire about information pertaining to all children with DKA. Participating clinics completed a retrospective chart according to the medical records of the children. The International Society for Pediatric and Adolescent Diabetes (ISPAD) defined DKA as hyperglycemia (blood glucose > 11 mmol/L or 200 mg/dl) and blood pH < 7.3, or bicarbonate < 15 mmol/L and ketonemia or ketonuria. The severity of DKA (mild, moderate, severe) is classified according to the ISPAD guidelines (17). For the definition of severity, mild DKA is a venous pH of 7.2–7.29 or bicarbonate < 15 mmol/l, moderate DKA is a venous pH of 7.1–7.19 or bicarbonate < 10 mmol/l, and severe DKA is a venous pH of <7.1 or serum bicarbonate < 5 mmol/l, at admission (18). We excluded patients with type 2 diabetes from the study.

The medical records of all children who were diagnosed with DKA in the PICU were analyzed. The patients' medical histories were evaluated to determine whether DM was newly diagnosed and to determine the time from diagnosis to DKA. Age, sex, family history of diabetes in first- and/or second-degree relatives, body mass index, the date of admission to the pediatric intensive care unit, complications, time to take crystallized insulin, and the length of stay in pediatric intensive care units were recorded. Mortality scoring systems are widely used in pediatric intensive care units. The most well-known and frequently used Pediatric Risk of Mortality (PRISM) score and for the assessment of the level of consciousness the Glasgow Coma Scale (GCS) were also noted.

The data on serum biochemistry included serum glucose, sodium, potassium, blood urea nitrogen, creatinine, blood and urine ketone measurements, and hemoglobin A1c (HbA1c); venous blood gas analysis (pH, HCO_3_, CO_2_, and lactate levels), complete blood count analysis [white blood cell count (WBC)], absolute neutrophil count (ANC), and absolute lymphocyte count were recorded.

The primary objective of this study was to evaluate the number of children admitted to the PICU with DKA and to compare disease characteristics, clinical findings, the severity of DKA, and laboratory features between the pre-pandemic period and the first year of the COVID-19 pandemic. The secondary objective of this study was to evaluate children with new-onset Type 1 diabetes presenting with DKA and to compare disease characteristics, clinical findings, the severity of DKA, and laboratory features between the pre-pandemic period and the first year of the COVID-19 pandemic. Another secondary objective was to evaluate the demographic, clinical, and laboratory features of children with DKA, as well as the positive polymerase chain reaction (PCR) test for SARS-CoV-2 or acute COVID-19 infection or Multisystem inflammatory syndrome in children (MIS-C), when available.

### Statistical Analysis

Continuous data are represented as median and interquartile range IQR (25–75%) and frequencies (%) for categorical variables were calculated. The distribution of the quantitative variables in the analyzed sample was compared with the normal distribution using the Shapiro–Wilk test. If the data were normally distributed, a two-tailed unpaired *t*-test was used to compare the continuous variables between groups. A non-parametric Mann–Whitney U test was performed to compare results between groups for non-normally distributed data. Fisher's exact test or chi-square tests (the non-parametric *x*^2^ and Kruskal–Wallis tests) of association were applied to assess whether any differences existed between each categorical factor and each binary outcome. The difference in the rate of severe DKA and new-onset DKA between the pre-pandemic year and the first year of the COVID-19 pandemic were analyzed using the chi-square test or Fisher's exact test, where the assumptions of the chi-square test are not met. A *p*-value of <0.05 was used to determine statistical significance. Statistical analysis was completed using SPSS for Windows.

## Results

We obtained and analyzed data from 997 (448; 44.9% male) children and adolescents with DKA-−517 before the pandemic and 480 during the pandemic period—who were diagnosed from March 1, 2019 to February 28, 2021, from 27 pediatric intensive care units in Turkey. 589 (59%) of these patients presenting with DKA had new-onset diabetes Type 1.

### Comparison Between the First Year of the COVID-19 Pandemic and the Pre-pandemic Period

**Figure 1:**
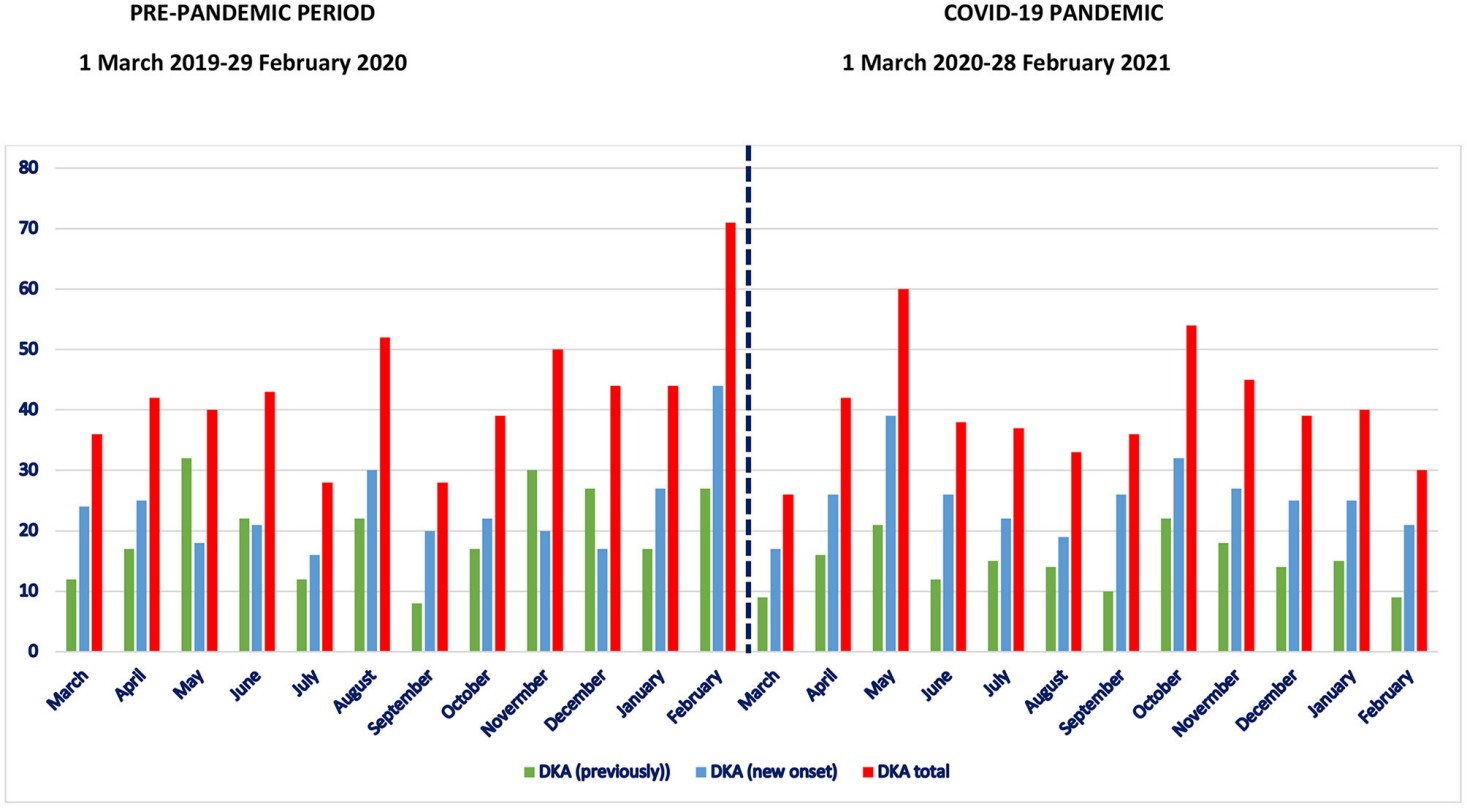
Number of children with diabetic ketoacidosis (red), number of previously type 1 diabetic children with DKA (green) and DKA among children with new onset type 1 diabetes (blue) during the pre-pandemic and COVID-19 pandemic period.

While the number of DKA admissions to the PICU was slightly lower during the COVID-19 pandemic (*n* = 480) compared to the pre-pandemic period (*n* = 517), the newly diagnosed DKA was 54.9% in the pre-pandemic period and 63.5% in the COVID-19 pandemic period (*p* < 0.01) (Table 1; Figure 1). The most frequent pediatric intensive care admissions were in February before the pandemic and in May 2020 and October–December 2020 during the COVID-19 pandemic period (Figure 1). Age and gender distributions were similar between the two study periods (*p* > 0.05). The most common symptoms at presentation were vomiting, polyuria, polydipsia, weakness, and rapid breathing in both the pre-pandemic and pandemic periods (*p* > 0.05). During the COVID-19 pandemic period, there was a significantly higher rate of DKA amongst those without a first-degree relative with DM (75.6 vs. 65.8., *p* < 0.05). The incidence of severe DKA was also significantly higher compared with the pre-pandemic period (70.2% in the COVID-19 period vs. 56.5% in the pre-pandemic period; *p* < 0.0001). The PRISM score was also significantly higher in the pandemic period (*p* < 0.0001) (Table 1). There are no difference for age of onset of diabetes between pre-pandemic and pandemic period (*p* > 0.05). The body mass index (BMI) of the children who had to be admitted to the PICU due to DKA before and during the pandemic were similar (*p* > 0.05).

**Table 1:** Clinical features of children with DKA during pre-pandemic and COVID-19 pandemic period.

	**Pre-pandemic *n* = 517**	**COVID-19 pandemic *n* = 480**	***p-*value**
Gender (Boys)	241 (46.6%)	207 (43.1%)	ns
Age (months)	133 (93.0–168)	130 (84.0–168)	ns
New Onset DM (*n*/%)	284 (54.9%)	305 (63.5%)	***p*** **<** **0.01**
Previously DM (*n*/%)	233 (45.1%)	175 (36.5%)	***p*** **<** **0.01**
Family history of DM (*n*/%)	156 (30.2%)	117 (24.4%)	***p*** **<** **0.05**
Severe DKA	292 (56.5%)	337 (70.2%)	***p*** **<** **0.0001**
Moderate DKA	130 (25.1%)	98 (20.4%)	ns
Mild DKA	95 (18.4%)	45 (9.4%)	***p*** **<** **0.0001**
PRISM score	8.00 (6.00–12.0)	11.0 (9.00–13.0)	**<0.001**
GCS score	13.0 (12.0–15.0)	13.0 (12.0–15.0)	ns
GCS: Moderate-Severe (<14)	281 (54.3%)	256 (53.4%)	ns
Duration of insulin infusion (h)	17.0 (12.0–23.0)	18.0 (12.0–24.0)	***p*** **<** **0.05**
Presence of complication (*n*/%)	378 (73.1%)	329 (68.5%)	ns
Use of antibiotic (n/%)	114(22.1%)	162(33.8)	**<0.001**
Duration of PICU stay (hours)	24.0 (16.0–28.0)	24.0 (20.0–43.3)	**<0.001**

*All data are represented as median and interquartile range IQR (25–75%). ns, Not significant. Statistically significant values showed as bold*.

Serum glucose levels at admission were similar between the two groups (*p* > 0.05). At admission, serum pH (*p* < 0.001), HCO_3_ (*p* < 0.05), and CO_2_ (*p* < 0.05) levels were significantly lower in children with DKA during the COVID-19 pandemic period (Table 2). Serum HbA1c levels were also higher during the pandemic period (*p* < 0.0001). There was a significant increase in the duration of insulin infusion and length of PICU stay during the COVID-19 pandemic (*p* < 0.05 and *p* < 0.0001, consecutively) (Table 1). The use of antibiotics in the PICU was also significantly higher during the pandemic period (*p* < 0.001). There were no mortalities during the study period. However, a renal replacement therapy requirement occurred in one patient before the pandemic and in three patients during the pandemic period. Brain oedema was detected in two patients before the pandemic and in six patients during the pandemic period. The clinical and laboratory features of children with previously diagnosed DM and new-onset Type 1 diabetes are shown in Tables 3, 4. We evaluated the risk factors for DKA among children with previously T1DM. Among these 175 children, 68.5% (*n* = 120) out of them misses their regular insulin doses, 58.8% (*n* = 103) did not regularly recorded their blood glucose levels and 54.2% (95) had low dietary compliance.

**Table 2:** Laboratory features of children with DKA during pre-pandemic and COVID-19 pandemic period.

	**Pre-pandemic *n* = 517**	**COVID-19 pandemic *N* = 480**	***p*-value**
pH	7.09 (6.97–7.19)	7.03 (6.91–7.14)	***p*** **<** **0.001**
HCO3	7.00 (4.80–10.0)	6.30 (4.90–8.63)	***p*** **<** **0.01**
pCO2	20.2 (16.0–25.0)	19.8 (16.0–24.5)	***p*** **<** **0.05**
Lactate	1.99 (1.35–2.80)	2.00 (1.41–2.90)	ns
Serum glucose (mg/dl)	469 (371–583)	467 (388–568)	ns
HbA1c	11.7 (10.1–13.5)	12.4 (10.9–14.0)	***p*** **<** **0.001**
Sodium	132 (130–136)	133 (130–136)	***p*** **<** **0.05**
Potassium	4.40 (3.84–4.90)	4.23 (3.80–4.86)	ns
Blood urea nitrogene	19.0 (12.0–32.0)	17.0 (12.0–27.0)	**<0.01**
Creatinine	0.8 (0.6–1.0)	0.75 (0.6–1.0)	**<0.01**
Phosphorus	3.60 (2.90–4.41)	3.56 (2.80–4.34)	ns
WBC	14,900 (9,860–22,900)	17,000 (11,600–24,400)	**<0.01**
Absolute neutrophilc count (mm^3^)	8,600 (4,690–15,300)	10,900 (5,790–17,800)	***p*** **<** **0.001**
Absloute lymphocyte count (mm^3^)	3,020 (2,030–4,700)	3,060 (2,110–5,200)	ns

*All data are represented as median and interquartile range IQR (25–75%). ns, Not significant. Statistically significant values showed as bold*.

**Table 3:** Clinical features of new onset type 1 diabetic children with DKA during pre-pandemic and COVID-19 pandemic period.

	**New onset type 1 diabetes**			**Previously type 1 diabetes**		
	**Pre-pandemic (*n* = 284)**	**COVID-19 pandemic (*n* = 305)**	***p*-value**	**Pre-pandemic (*n* = 233)**	**COVID-19 pandemic (*n* = 175)**	***p*-value**
Gender (Boys)	140 (49.2%)	148 (48.5%)	ns	101 (43.3%)	59 (33.7%)	ns
Age (months)	115 (57.5–149)	100 (52.0–141)	ns	158 (131–182)	163 (138–183)	ns
Family history of DM (n/%)	82 (28.8%)	77 (25.2%)	ns	73 (31.3%)	40 (22.9%)	ns
Duration of symptoms (days)	7.00 (3.00–15.0)	7.00 (3.00–14.0)	ns	1.00 (1.00–3.0)	2.00 (1.00–3.0)	ns
Severe DKA	165 (58%)	226 (74.0%)	***p*** **<** **0.0001**	127 (54.5%)	111 (63.4%)	ns
Moderate DKA	60 (21.12%)	53 (17.3%)	ns	70 (30.0%)	45 (25.7%)	ns
Mild DKA	59 (20.7%)	26 (8.5%)	***p*** **<** **0.0001**	36 (15.5%)	19 (10.9%)	ns
PRISM score	8.00 (6.00–12.0)	11.0 (10.0–13.0)	***p*** **<** **0.001**	9.00 (6.00–12.0)	11.0 (8.00–12.0)	ns
GCS score	13.0 (12.0–15.0)	13.0 (12.0–15.0)	ns	13.0 (12.0–15.0)	14.0 (12.0–15.0)	ns
Duration of insulin infusion (h)	18.0 (14.0–24.0)	20.0 (14.0–25.0)	ns	15.0 (12.0–20.0)	14.0 (11.0–22.0)	ns
Presence of complication (n/%)	197 (69.3%)	195 (63.9%)	ns	180 (77.3%)	134 (76.6%)	ns
Use of antibiotic (n/%)	52(18.3%)	94(30.8%)	*p* < 0.001	62(26.6%)	68(38.9%)	***p*** **<** **0.05**
Duration of PICU stay (hours)	24.0 (20.0–34.0)	24.0 (20.0–47.0)	***p*** **<** **0.01**	20.0 (14.0–24.0)	24.0 (16.0–36.0)	***p*** **<** **0.01**

*Statistically significant values showed as bold*.

**Table 4:** Laboratory features of new onset type 1 diabetic children with DKA during pre-pandemic and COVID-19 pandemic period.

	**New onset type 1 diabetes**			**Previously type 1 diabetes**		
	**Pre-pandemic (*n* = 284)**	**COVID-19 pandemic (*n* = 305)**	***p*-value**	**Pre-pandemic (*n* = 233)**	**COVID-19 pandemic (*n* = 175)**	***p*-value**
pH	7.09 (6.95–7.19)	7.01 (6.91–7.12)	***p*** **<** **0.001**	7.10 (6.99–7.19)	7.05 (6.93–7.16)	***p*** **<** **0.01**
HCO3	6.30 (4.50–10.0)	6.10 (4.60–8.00)	ns	7.40 (5.30–10.0)	6.90 (5.20–9.75)	ns
pCO2	19.6 (15.4–25.0)	19.0 (15.2–22.7)	ns	21.5 (17.0–26.0)	21.0 (16.0–27.0)	ns
Lactate	1.71 (1.23–2.48)	1.90 (1.40–2.70)	ns	1.71 (1.23–2.48)	1.90 (1.40–2.70)	ns
Serum glucose (mg/dl)	470 (377–574)	471 (400–566)	ns	467 (369–590)	456 (370–572)	ns
HbA1c	11.7 (10.2–13.3)	12.4 (10.9–14.0)	***p*** **<** **0.001**	11.9 (10.2–14)	12.2 (10.8–14.0)	ns
Sodium	132 (129–135)	132 (130–135)	ns	133 (130–136)	134 (131–136)	ns
Potassium	4.08 (3.60–4.50)	4.04 (3.61–4.43)	ns	4.80 (4.34–5.30)	4.80 (4.20–5.31)	ns
Blood Urea Nitrogene	16.0 (11.0–28.0)	14.0 (10.0–23.0)	ns	23.0 (16.0–37.0)	21.0 (14.5–33.0)	ns
Creatinine	0.7 (0.6–0.9)	0.7 (0.5–0.9)	ns	0.9 (0.7–1.1)	0.9 (0.7–1.09)	ns
Phosphorus	3.40 (2.79–4.00)	4.04 (3.61–4.43)	ns	3.99 (3.00–5.01)	4.80 (3.00–5.48)	ns
WBC	14,300 (9,500–22,000)	15,900 (11,500–23,000)	***p*** **<** **0.05**	16,000 (10,300–23,900)	19,000 (12,700–26,600)	***p*** **<** **0.01**
Absolute neutrophil count (mm3)	7,400 (4,020–13,300)	9,600 (5,610–16,600)	***p*** **<** **0.001**	10,300 (5,560–17,900)	13,500 (6,780–19,700)	ns
Absolute lymphocyte count (mm3)	3,250 (2,220–5,950)	3,180 (2,110–5,540)	ns	2,690 (1,840–3,860)	2,760 (2,080–4,650)	ns

*Statistically significant values showed as bold*.

### Comparison Between the First Year of the COVID-19 Pandemic and the Pre-pandemic Period Among Children With New-Onset Type 1 Diabetes

The newly diagnosed Type 1 diabetes presenting with DKA was 54.9% in the pre-pandemic period and 63.5% in the COVID-19 pandemic period (*p* < 0.0001). The mean age at presentation and gender distribution were similar between both groups (*p* > 0.05) (Table 3). The duration of symptoms was not different for either period in patients with new-onset type 1 DM (*p* > 0.05). The PRISM score at admission, in the new-onset Type 1 diabetes group, was also significantly higher in the COVID-19 pandemic period (*p* < 0.001). Serum glucose concentrations at admission were similar between the two time periods (*p* > 0.05). The mean pH and bicarbonate levels at presentation were significantly lower during the COVID-19 pandemic period compared to the pre-pandemic period (*p* < 0.001 and *p* < 0.01, respectively). The incidence of severe DKA was also higher during the COVID-19 pandemic period [among children with new onset Type 1 diabetes (74.0 vs. 58.3%; *p* < 0.0001)]. HbA1c levels were also higher during the COVID-19 pandemic period (*p* < 0.001). Furthermore, the length of PICU stay in patients with new-onset Type 1 diabetes was significantly longer during the COVID-19 pandemic period (36.4 ± 31.5 vs. 29.5 ± 18.7 h *p* < 0.01).

### Clinical and Laboratory Findings of Children With DKA and COVID-19 Infection

Eleven patients (aged between 32 and 216 months; three boys and eight girls) during the COVID-19 pandemic period tested positive for SARS-CoV-2 by reverse transcriptase polymerase chain reaction. Three out of 11 children were admitted at the first 6 months of the pandemic (March to August 2020), and eight were admitted for the following 6 months (September 2020 to February 2021). Eight out of 11 patients (72.7%) were diagnosed with new-onset Type 1 diabetes, and only one child had a family history of DM. Serum glucose levels varied between 367 and 800 mg/dl. Nine children had severe DKA (81.8%), and eight out of the nine children had pH levels lower than 7.0. Ten out of 11 children received antibiotic treatment. Only one 16-year-old girl with respiratory findings received favipravir for acute COVID-19 infection. None of the children had complications related to COVID-19 infection. Furthermore, there were no indications of multi-inflammatory syndrome in children (MIS-C) among the patients with DKA.

## Discussion

This is the first country-based study to look at DKA in children required to be admitted to the PICU during the first year of the pandemic in Turkey. The number of cases included in our analysis makes this study one of the largest DKA studies in the literature, spanning a full year of the pandemic. During the COVID-19 pandemic, we found a considerable increase in the severity of DKA. In children presenting with DKA, the percentage of new-onset type 1 diabetes was greater during the pandemic (63.5 vs. 54.9%). Severe DKA was considerably more common during the COVID-19 pandemic (70.2 vs. 56.5%) among children with new-onset Type 1 diabetes (74.0 vs. 58.3%).

Recent investigations showed an increase in Type 1 diabetic adolescents with DKA during the initial phase of the COVID-19 pandemic (7, 13–16). A German study found an increase in DKA and severe DKA between March and May 2020. The rate of DKA in children (44.7%) was much greater in 2020 than in the previous two years, with the risk of DKA being 1.84 times higher in 2020 than in 2019 (13). The incidence of Type 1 diabetes in Alberta, Canada, did not change throughout the COVID-19 pandemic; however, there was an increase in the incidence of DKA onset of Type 1 diabetes (68.2 vs. 45.6%; absolute increase of 22.6%) and severe DKA (27.1% in 2020 vs. 13.2% in 2019; absolute increase of 13.9%) (14). During the COVID-19 pandemic (March to May 2020), Lawrence et al. (15) observed a considerable rise in the frequency of severe DKA among children and adolescents (45 vs. 5%; absolute increase of 16.7) at new Type 1 diabetes diagnosis in Australia. Dyzgalo et al. (17) reported a modest rise in DKA incidence in 2020 compared to 2019, but severe DKA was more common (61.1 vs. 28.6%). Early pandemic study showed an increase in DKA admissions and severe DKA in children. During the first 12 months of the pandemic, we also observed an increase in DKA hospitalizations and severe DKA among newly diagnosed and all Type 1 diabetic children. During the COVID-19 pandemic, HbA1c levels, PRISM scores, insulin infusion times, and PICU stays were higher/longer in the entire research group and in children with newly diagnosed Type 1 diabetes, in Turkey. During the pandemic, reduced access to primary care, parental concern, and delayed Type 1 diabetes diagnosis may all contribute to greater DKA severity. Reduced pediatric admission to the pediatric emergency care unit due to respiratory tract infections or trauma may diminish incidental hyperglycemia recognition in Type 1 diabetic children.

Recent research into the occurrence of Type 1 diabetes during the COVID19 pandemic has yielded conflicting results. Since 2011, there has been an increase in the incidence of Type 1 diabetes in Germany; nevertheless, their research did not demonstrate any pandemic-related short-term changes in DM1 incidence between 2019 and 2020 (19). When compared to estimates of past Type 1 diabetes instances in the 5 years prior in the United Kingdom, Unsworth et al. (20) found an increase (80% increase) in new Type 1 diabetes cases between March 23, 2020 and June 4, 2020 and they considered SARS-CoV-2 to be a possible cause of new-onset Type 1 diabetes. Rabbone et al. (21) performed a cross-sectional electronic survey of all 68 Italian pediatric diabetes centers for their DKA patients, between February 2020 and April 2020, and showed a 23% lower diagnosis of Type 1 diabetes than in 2019; they explained that the lower number of new-onset diabetes cases might be due to lower exposure to seasonal viruses related to school closures. In our study, we collected the data of children presenting with DKA however, we did not have a chance to evaluate the incidence of children with newly diagnosed Type 1 diabetes.

According to recent meta-analyses, diabetes increases the risk of symptomatic SARS-CoV-2 infection and COVID-19-related hospitalization, escalation of care, invasive assisted mechanical ventilation, renal replacement therapy, cardiac injury, thromboembolic events, and death in adults with Type 2 diabetes (22). However, there is no evidence that children with well-controlled Type 1 diabetes are at a greater risk of COVID-19 infection. COVID-19 and other viral infections can lead to significant consequences in persons with diabetes, such as DKA. Adults and children can develop new-onset diabetes as a result of COVID-19 infection; however, there have only been a few case reports on this matter so far (23–31). In our study, during the COVID-19 pandemic, 11 children with DKA (ages 32 to 216 months; three boys and eight girls) tested positive for SARS-CoV-2) at admission. Of these children, 72.7% developed Type 1 diabetes for the first time, and 81.8% had severe DKA. There were no COVID-19-related problems in any of the children. In our study, there were no children with DKA who had MIS-C. Between March and July 2020, 2.9 percent of COVID-19-related pediatric hospitalizations in 14 U.S. states had newly diagnosed DKA (12). In a multicenter study from the UK, five children with positive results (two tested were SARS-CoV-2 PCR positive and three were SARS-CoV-2 IgG positive) presented with severe DKA; three presented with severe DKA and refractory hypokalemia, and one PCR-positive child suffered a hypokalemia-related cardiac arrest but fully recovered (20). Eight patients were diagnosed with COVID-19 in Italy between February 20 and April 14, 2020 (aged between 6 and 16 years), one of whom was new onset, and all of whom were asymptomatic or had only mild symptoms (28). Hawkes et al. (16) found 73 cases of newly diagnosed T1D between March 16 and July 31, 2020, of which two (a 3-year-old girl and a 9-year-old boy) were positive with mild disease. Guemes et al. (27) found that the severity of new-onset Type 1 diabetes in the pandemic group, which included 10 children, two of whom tested positive for SARS CoV-2 between March 21 and May 6, 2020, was considerably greater. Because two of these patients had minimal respiratory symptoms, no special SARS-CoV-2 therapy was necessary. A previously healthy seven-year-old with asymptomatic COVID-19 presented with a high nasopharyngeal viral load, detectable COVID-19 IgG antibodies, DKA, and islet cell autoantibodies, according to Nielsen-Saines et al. (28). Another example reported is a 19-year-old with new-onset DKA (autoantibody negative) after COVID-19 (29). Rabizadeh et al. (30) describe a 16-year-old child with recently diagnosed diabetes who developed severe DKA, acute kidney injury, and COVID-19 infection as the first manifestation of his diabetes. Naguib et al. (31) first described new-onset diabetes with DKA in an 8-year-old girl with COVID-19-induced multisystem inflammatory disease It is crucial to look into the possibility of a link between COVID-19 and Type 1 diabetes in youths. The number of instances of T1DM in children and adolescents increased during the COVID-19 pandemic, with evidence pointing to a link between the two diseases. COVID-19 infection has also been suggested as a possible cause of ketoacidosis by causing direct damage to pancreatic beta cells. It is difficult to tell whether new-onset instances of DM1 are caused by viral harm or by COVID-19-induced immunological dysregulation. While DM is a risk factor for severe COVID-19, Type 1 diabetes is also caused by SARS-CoV-2 infection. It's uncertain whether pancreatic damage is directly caused by the virus's cytopathic effect or indirectly caused by a high systemic inflammatory response and multiorgan failure in severe COVID-19 disease. COVID-19-associated diabetes results from chronic disease susceptibility and COVID-19-specific metabolic pathways, not a single event (32). During the study period, no COVID-19 vaccine has been registered for children and adolescents in Turkey, and none of our study population have been vaccinated.

Our study has some limitations. Because of the retrospective character of our study, it is prone to miss data and is limited by the incomplete medical records and the quality of parents' or caregivers' medical history reports. Blood osmolality could not be evaluated as it was not routinely measured in most centers. We did not collect data on newly diagnosed Type 1 diabetic patients who did not need to be admitted to the PICU. SARS-CoV-2 antigen testing was favored by certain centers, but we did not have SARS-CoV-2 antigen testing data for all children. Furthermore, antibodies against SARS-CoV-2 were not tested. Regarding to hospitalization criteria for DKA varies between PICUs, however there are no changes for DKA protocol for participating centers between pre-pandemic and pandemic period.

The study's main findings include a high frequency of children with DKA at diagnosis and an initially increased trend in DKA severity at Type 1 diabetes diagnosis in Turkey during the first year of the COVID-19 pandemic. Severe DKA is not only life-threatening, but it also necessitates the use of intensive care beds and resources during a time when demand is likely to be high (15). The impact of the SARS-CoV-2 virus, the COVID-19 pandemic, the Stringency Index, and non-pharmaceutical measures on the incidence of Type 1 diabetes in children is still unknown. The observed rise in the number of juvenile patients admitted with DKA could be explained by the indirect effects of COVID-19, but the direct effect of SARS-CoV-2 on diabetes etiology should be investigated. Healthy child follow-up, routine immunization practices, follow-up of chronic disorders, and acute complications have all been demonstrated as being affected during pandemic periods as a result of parents' fears about their children becoming sick. Lessons learned during the COVID-19 lockdown, highlight the importance of raising physician and public awareness about the signs and symptoms of Type 1 diabetes, as well as the importance of considering diabetes as a possible diagnosis in those with non-specific symptoms (17). Factors such as school closures, difficulty adapting to a new diet owing to a constant stay at home, limited exercise, and stress in the child and family can all contribute to diabetes control challenges. It will be critical to assess the impact of school closures, quarantine, and a lack of exposure to seasonal infections on the rate of future diabetes diagnoses (15, 21). During a worldwide pandemic, children and their families must also be encouraged to seek and get healthcare for non-pandemic-related health concerns. Since the end of 2019, COVID-19 vaccinations have been accessible, primarily for adult immunization, in Turkey and around the world. The reduction in respiratory tract infections and other communicable diseases during school closure and national lockdown, according to Raucci et al. (33) should cause us to consider the potential impact of these conditions on the health system when schools reopen. Vaccines' effects on the circulating virus could help alleviate pandemic constraints. More research is needed to better understand the impact of viral and pandemic-related constraints on the occurrence of Type 1 diabetes and its sequelae.

## Data Availability Statement

The raw data supporting the conclusions of this article will be made available by the authors, without undue reservation.

## Ethics Statement

The studies involving human participants were reviewed and approved by Eskisehir Osmangazi University. Written informed consent from the participants' legal guardian was not required to participate in this study in accordance with the national legislation and the institutional requirements.

## Author Contributions

EKi, BK, and ED developped the concept and wrote the manuscript. MBi analyzed the data for statistical evaluation. ED is the senior author for this manuscript. EKi is the guarantor of this work. All authors reviewed, edited, and approved the final version of the manuscript.

## Conflict of Interest

The authors declare that the research was conducted in the absence of any commercial or financial relationships that could be construed as a potential conflict of interest.

## Publisher's Note

All claims expressed in this article are solely those of the authors and do not necessarily represent those of their affiliated organizations, or those of the publisher, the editors and the reviewers. Any product that may be evaluated in this article, or claim that may be made by its manufacturer, is not guaranteed or endorsed by the publisher.
